# Persistence of the Knee-Jerks in Tabes Dorsalis

**Published:** 1910-07-09

**Authors:** 


### Medicine.

### PERSISTENCE OF THE KNEE-JERKS IN TABES DORSALIS.

In the case of locomotor ataxy (tabes dorsalis) the knee-jerks are so frequently absent that there is considerable hesitancy on the part of most practitioners to diagnose that disease when the knee-jerks are still present. Nevertheless there are many cases in which locomotor ataxy is the correct diagnosis and yet the knee-jerks are present.

This fact has been insisted upon by a great many neurologists, though there may naturally be a feeling in the minds of many that the diagnosis may in some of these cases at least prove to have been incorrect; hence the following case in which the knee-jerks were present during life, and in which the nature of the disease was confirmed by post-mortem examination, is of interest. Death was due to tuberculosis, aortic regurgitation, and suppurative pyelonephritis.

The patient was a man aged fifty-six, a stonemason, who came under observation on July 16. At that time his mental condition prevented him from giving any very clear account of himself or of his family, but it appeared he had been a soldier and had lived abroad, and had there suffered from intermittent fever and also from a chancre, which had left little or no scar, and which had been followed by comparatively slight secondary symptoms. Apparently his locomotor ataxy dated back at least ten years, for at that time he had a perforating ulcer on the left foot, which got well only after prolonged treatment. About the same time he also experienced lightning pains which came on in paroxysms and prevented him from sleeping at night, and these had occurred at intervals on and off since. He had also had diplopia at that time; that is to say, ten years before his admission. From then onwards he had from time to time been laid up in bed, and had been a frequent inmate of various hospitals. Latterly he had suffered from girdle pains and from pains in his upper limbs similar to those in his legs. Bladder symptoms had been a prominent feature of the case almost from its beginning; they had been very troublesome and presented varying degrees of incontinence of the urine, of retention of the urine, and of dysuria. There had also been visceral crises, of which the most striking had been gastric; these set in two years before his final admission. He had also had acute rectal crises accompanied by attacks of piles.

When seen on July 16 the patient looked older than he really was owing to the whiteness of his hair, and he seemed thin and old with a cachectic, almost cancerous, facies. His main complaint was his bladder. He was obliged to adopt all sorts of attitudes in order to micturate and the passage of his urine caused him much pain. There was at this time retention with overflow, and the urine contained both blood and pus. The patient's liver and spleen were both enlarged, the heart presented the typical signs of aortic regurgitation, and there were apical râles suggestive of phthisis, the latter diagnosis being confirmed by the discovery of tubercle bacilli in the sputum. The pupils were of the Argyll-Robertson type, reacting fairly well to accommodation, but not to light. The patient could walk, but with typical tabetic gait. Romberg's sign was present, but not very marked, and when the patient was lying in bed inco-ordination was not very obvious. The perforated ulcer on his foot was quite healed.

The knee-jerks were examined on many occasions, and they were always present and of normal strength without any diminution or inequality. The plantar reflexes were flexor. At night there was now delirium of a low muttering type; catheterisation was necessary; but in spite of treatment the patient sank and died partly from uræmic trouble and partly from exhaustion from his heart and lung disease. At the post-mortem examination the diagnosis of phthisis was confirmed; the heart weighed 560 grams, and there was aortic regurgitation due to syphilitic aortitis. The kidneys, the ureters, and the bladder were in a state of acute pyelonephritic inflammation. The brain looked perfectly healthy and was of normal weight. The spinal cord was obviously diminished in volume, and even the naked eye could detect atrophy of the posterior columns and roots.

The histological examination confirmed these spinal cord changes. The essential lesions were in the posterior roots and columns; they varied much in the different regions of the cord, being most marked in the sacral and lower lumbar regions, less marked in the upper lumbar and lower dorsal, and more marked again in the upper dorsal and in the cervical regions. The changes were typical of tabes dorsalis.

How, then, can the preservation of the knee-jerks here be accounted for?

Normally the sensori-motor reflex path upon which the knee-jerk depends—even though it is not in the strict sense of the word a true reflex—consists of the posterior nerve roots, the ending of these fibres in the cord, the connecting fibres between these and the anterior cornual cells, the latter cells themselves in the upper part of the lumbar enlargement, the fibres issuing from them in the anterior nerve roots and passing thence to the anterior crural nerve supplying the quadriceps extensor femoris muscle; if any part of this arc is broken, the knee-jerk disappears. Such destruction as a rule occurs in locomotor ataxy owing to the lesions in the posterior nerve roots and in the cord itself in the region of the point of entry of the posterior nerve roots. If, then, the loss of knee-jerks is typical in tabes, why can they in exceptional cases persist?

### A Probable Explanation.

In certain cases of locomotor ataxy the knee-jerk having been abolished for a longer or shorter time, has reappeared as a result of some cerebral complication. It has been supposed, therefore, that the abolition of the knee-jerk in these cases of tabes is due to changes in the cord itself rather than in the posterior nerve roots; and that when the cerebral lesion occurs the cortical inhibition of the spinal centres is removed so that the medullary reflex activity becomes sufficiently increased again to bring the knee-jerks back.

This explanation does not, however, cover those cases in which the knee-jerks have been retained without ever having disappeared at all. Westphal has shown that there exists in the upper part of the lumbar enlargement of the spinal cord a region the destruction of which determines the disappearance of the knee-jerks. The most important part of this region is that to which the term posterior root zone has been applied, and it has been stated by some authorities that whenever the knee-jerks have been preserved this posterior root zone has remained intact in the upper part of the lumbar enlargement. There have even been cases in which the knee-jerk has been retained on one side and lost on the other, the absence of the corresponding posterior root zone and the presence of the other being confirmed by post-mortem examination.

In the case described above the posterior root zones had escaped injury on both sides of the lumbar enlargement.

Cases of tabes with preservation of the knee-jerks are by no means exceptional; though, as one might expect, in proportion as the disease progresses the knee-jerks tend to disappear.

